# The Influence of Socioeconomic Factors on Access to Biologics in Psoriasis

**DOI:** 10.3390/jcm12237234

**Published:** 2023-11-22

**Authors:** Jenny M. Norlin, Sofia Löfvendahl, Marcus Schmitt-Egenolf

**Affiliations:** 1 The Swedish Institute for Health Economics (IHE), SE-223 61 Lund, Sweden; jenny.norlin@ihe.se (J.M.N.); sofia.lofvendahl@ihe.se (S.L.); 2 Department of Public Health and Clinical Medicine, Dermatology, Umeå University, SE-901 85 Umeå, Sweden; 3 Centre for Pharmacoepidemiology, Karolinska Institutet, SE-171 77 Stockholm, Sweden

**Keywords:** access, psoriasis, PASI, DLQI, biologics, socioeconomic

## Abstract

Background: Since the introduction of biologics for psoriasis, uptake has been uneven and limited. Few studies have investigated the influence of socioeconomic factors on access to biologics. Objective: To investigate how socioeconomic factors influenced access to biologics. Methods: Biologic-naïve patients in the Swedish National Register for Systemic Treatment of Psoriasis (PsoReg) for the years 2006–2014 were included. For patients who remained on nonbiologic treatments during their entire registration (*n* = 1851), the most recent registration was analyzed. For patients who began treatment with biologics during registration in PsoReg (*n* = 665), the last observation before initiation of biologics was analyzed. A logistic regression model was used to investigate whether education and income influenced the probability of a switch to biologics, whilst adjusting for demographic and individual factors such as age, sex, disease severity, and clinical characteristics. Results: The odds ratio of access to biologics was 1.8 (CI = 1.3–2.6) in the group with a high level of disposable income, compared with the middle-income group. No differences were found concerning educational levels. The odds ratios of access to biologics decreased with age. Patients with psoriatic arthritis had odds ratios of access to biologics which were more than 50 percent higher, controlling for other variables. High disease severity, in terms of physician- and patient-reported severity, increased the odds ratios of access to biologics. Conclusions: The higher-income group had better access to biologics than the middle-income group when adjusting for disease severity and lifestyle factors. This may not only be an equity problem, as a better allocation of society’s resources might have resulted in a higher overall effectiveness of biologics.

## 1. Introduction

Psoriasis is a chronic, immunological and systemic disease with a prevalence of around 3–4 percent. Psoriasis causes the skin cells to grow too quickly, resulting in raised, red, scaly patches on the skin that can be very itchy and painful. Psoriasis is associated with the joint disease psoriasis arthritis and with other comorbidities, including depression. There is no cure for the disease, but there are several treatment options available to manage the symptoms. Patients with moderate-to-severe psoriasis generally need systemic agents. A biologic systemic agent is a type of drug that is made from living cells and consists of proteins. Biologic drugs target specific parts of the immune system that cause inflammation in the body. Biologics work by targeting the specific proteins in the immune system that cause inflammation. By blocking these proteins, biologics can reduce the symptoms of psoriasis. Biologics have fewer side effects than traditional systemic medications used to treat psoriasis, such as methotrexate and cyclosporine.

When biologic treatment became available for moderate-to-severe psoriasis and psoriatic arthritis, it radically changed treatment outcomes and health-related quality of life (HRQoL) for patients with moderate-to-severe psoriasis. Moreover, biologics have even been shown to be life-saving in some cases of severe forms of pustular psoriasis [1].

Moderate-to-severe psoriasis was often defined as a Psoriasis Severity and Area Index (PASI) > 10 and/or a Dermatology Life Quality Index (DLQI) > 10. When biologics were introduced in Sweden, treatment guidelines and the reimbursement authority restricted the use of biologics to patients with moderate-to-severe psoriasis who did not respond to conventional systemic treatment or who exhibited intolerance or contraindications to conventional treatments.

Since the introduction of biologics in Sweden in 2004, access has been limited and uptake has been uneven. Previous observational studies carried out in Sweden have suggested that the use of eligibility criteria according to guidelines was a poor predictor of access to biologics [2,3]. These criteria combine the clinical measure PASI [4] and the health-related quality of life measure DLQI [5]. Several studies have investigated factors affecting access to biologics in a Swedish setting. Elderly people with psoriasis were found to be less likely to obtain access to biologics than younger people [6]. Men were found to be more likely to be prescribed biologics than women, but the observed difference was associated with higher disease severity among men [7]. Small or negligible differences in access to biologics have been found between different types of healthcare providers [8], whereas geographical differences between healthcare regions have been found to persist, after adjusting for disease severity [9]. Socioeconomic status factors are associated with healthcare disparities within [10,11] as well as between countries [12]. Among people with psoriasis in Sweden, lower- and higher-income groups consume more healthcare, compared with medium-income groups [13]. However, no studies have yet analyzed the influence of socioeconomic factors on access to biologics in psoriasis in a Swedish setting, and only a few have done so in a European setting [14,15]. In the first decade of biologics, no biosimilars or modern interleukin inhibitors were available. Consequently, with limited competition, TNF inhibitors had relatively high and stable acquisition costs. This enabled a straightforward analysis of the influence of socioeconomic factors on access to biologics.

The objective of this study was to investigate whether the socioeconomic factors of income and education exerted any influence which might explain some of the variation in the early access to biologics in patients with psoriasis in Sweden, when controlling for demographic and individual factors such as age, sex, and disease severity, along with other clinical characteristics.

## 2. Materials and Methods

### 2.1. Setting

In Sweden, healthcare is predominately publicly financed. Of the total cost, private insurance covers less than 1 percent, with 15 percent covered by patients themselves out of their own pockets. Healthcare in Sweden is also mainly publicly provided, by twenty-one regional authorities which are grouped into six healthcare regions in order to facilitate cooperation regarding specialized care. The regional authorities are responsible for the drug budget, but they also receive government contributions. The regional authorities collaborate at a national level, as well as at the level of the six large healthcare regions [16].

### 2.2. Materials

This study used data from the Swedish Register for Systemic Treatment of Psoriasis, or PsoReg [17]. Local, regional and university hospitals, as well as private clinics and hospitals and treatment centres managed by the patient organization PSO all participate in PsoReg. Data from PsoReg were extracted for the period from 2006, when the register was introduced, to 2014, when the register was linked to the Swedish longitudinal database on health insurance and labour market studies (LISA), using the Swedish personal identification number. Information about age, sex, disease severity and lifestyle factors were retrieved from PsoReg. Information on education and income was retrieved from LISA.

### 2.3. Included Patients and Observations

Patients with psoriasis who were bio-naïve at first registration in PsoReg were included. Observations were in line with clinical practice; they took place when patients visited their physician, and no visits were mandatory or protocol-driven. One observation per patient was used. For patients who remained on nonbiologic treatments during their entire registration, the most recent registration in PsoReg was used for analyses. For patients who began treatment with biologics during registration in PsoReg, the last observation prior to initiation of biologics was used for analyses. If both DLQI and PASI values were missing, the most recent observation in the previous 6 months was used. Similarly, if one of the DLQI or PASI values was missing, the most recent observation within the previous 6 months was carried forward. Outcomes registered more than 6 months prior to the switch were excluded. For each patient, the socioeconomic data for the year during which the included observation took place was used. The final dataset included observations made between 2006 and 2014. Patients who had used efalizumab (withdrawn in 2009) were excluded.

### 2.4. Explanatory Variables

Variables that were considered likely to affect the probability of being assigned biologics were included in a regression analysis. Age was included as a continuous variable. Male sex was used as a reference category. Disease severity was measured by PASI and DLQI. PASI measures the current severity of psoriasis based on coverage and level of severity in four body areas, resulting in a score from 0 (no disease) to 72 (theoretical maximal disease) [4]. The DLQI is a patient-reported questionnaire of ten questions on a four-point scale which relate to how the skin disease has affected patients’ quality of life over the previous week [5]. The score ranges from 0 (quality of life not impaired) to 30 (quality of life severely impaired). Moderate-to-severe psoriasis, and consequent eligibility for biologics, was defined as PASI ≥ 10 and/or DLQI ≥ 10. A categorical variable was therefore created to reflect these criteria. Four groups were included: (1) PASI < 10 and DLQI < 10; (2) PASI ≥ 10 and DLQI < 10; (3) PASI < 10 and DLQI ≥ 10; and (4) PASI ≥ 10 and DLQI ≥ 10. The reference category was PASI < 10 and DLQI < 10. Additional clinical variables included presence of psoriatic arthritis (PsA) or nail psoriasis. Lifestyle factors included BMI, smoking, and the high-risk use of alcohol. BMI was expressed in the following categories: (1) underweight (<18.5 kg); (2) normal weight (18.5 to 25 kg); (3) overweight (25 to 30 kg); and (4) obese (>30 kg). Normal weight was used as a reference category. In accordance with definitions issued by the Swedish National Board of Health and Welfare, high-risk use of alcohol was defined for both men and women as the consumption of more than 5 standard units of alcohol on a single occasion, or, for women only, 3–4 units at least four times. The no-risk use of alcohol and not smoking were used as reference categories.

Sweden is divided into six healthcare regions: North, Uppsala, Stockholm, Southeast, West, and South. Stockholm was used as the reference category. The year of the observation was included because access to biologics, in general, has increased over time, and observations for each patient may have occurred in different periods of time (2006–2008, 2009–2011, 2012–2014). The years 2009–2011 were used as the reference category. Education included the highest achieved level of education for each patient. Three levels were defined: “Low” = 0–9 years; “Medium” = 10–12 years; and “High” = 13 or more years. The “Medium” group was used as the reference category. Individualized disposable household incomes (including wages, transfers and taxes) were categorized into five income quintiles for all individuals in the study population. Incomes were inflation-adjusted to reflect the 2014 price level, using the consumer price index. The third income quintile was used as the reference category.

### 2.5. Statistical Analyses

Differences in patient characteristics were tested with t-tests for continuous variables, the chi-square test for categorical variables, and the Kruskal–Wallis test for outcome measures of disease severity. To identify potential factors associated with access to biologics, we carried out logistic regression using access to biologics (1 = access, 0 = no access) as the dependent variable. Odds ratios (ORs) and 95% confidence intervals (CIs) were estimated. 

As income and education are closely related, we performed two alternative regression analyses. The alternative models included the same variables as the main analyses, but one included only income and the other included only education. PsoReg includes patients with plaque psoriasis, as well as patients with uncommon clinical types of psoriasis, including erythrodermia, pustular psoriasis, and acrodermatitis continua suppurativa. As PASI is a tool developed for plaque psoriasis, a subgroup analysis was performed, where all patients with uncommon clinical types were excluded from the main analyses. 

Data analysis was performed using Stata Statistical Software Release 14 (StataCorp, College Station, TX, USA, 2015), StataCorp LP, R version 3.4.2 (R Core Team, R Foundation for Statistical Computing, Vienna, Austria, 2015) and statistical packages.

## 3. Results

The number of included patients was 2516; of these, 26 percent (*n* = 665) had received biologics and 74 percent (*n* = 1851) had received nonbiologic systemic treatments. Patient characteristics differed between biologically and nonbiologically treated patients (Table 1). Patients who received biologics were more likely to be young, male, and have plaque psoriasis, PsA or nail psoriasis. Patients who had access to biologics also had more severe psoriasis prior to biologic treatment, both clinically (PASI) and patient-reported (DLQI), compared with patients using nonbiologic agents. Biologically treated patients had higher BMIs than patients on nonbiologic systemic treatments, but were less likely to be smokers. Access to biologics also differed between geographical regions.

The year of inclusion differed between patient groups (*p* < 0.001). The year of inclusion was between 2006–2008 for 13 vs. 8 percent, between 2009–2011 for 40 vs. 40 percent and between 2012–2014 for 47 vs. 52 percent of patients on biologics and on nonbiologics, respectively.

**Figure 1:**
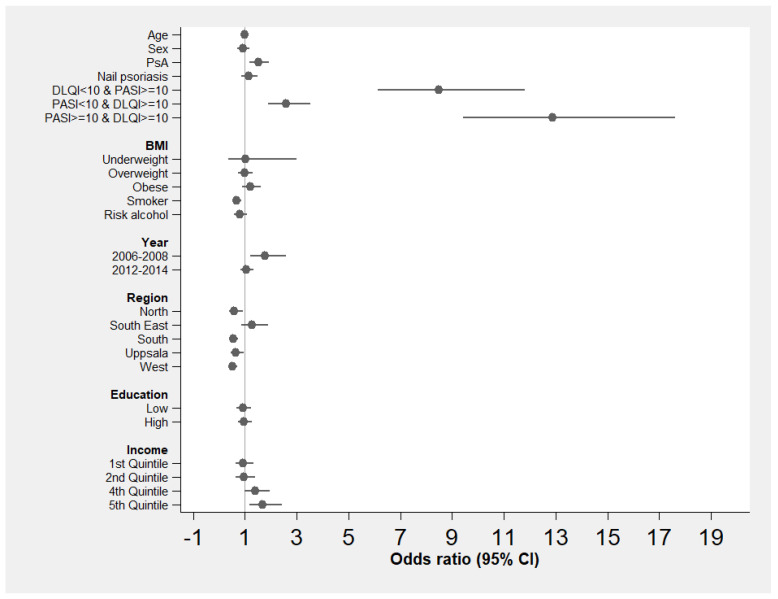
Odds ratios of access to biologics. BMI—body mass index; DLQI—Dermatology Life Quality Index; PASI—Psoriasis Area and Severity Index; PsA—psoriasis arthritis; Risk alcohol—high-risk consumption of alcohol.

The odds ratios of receiving biologics are presented in Figure 1. The lines around the point estimates show the 95% confidence intervals (CIs). The fourth and fifth quintiles of income were both statistically significant, compared with the reference group (third quintile); see Supplementary Materials, Table S1 and Figure 1. No differences were found in the other income groups. No differences were detected between different levels of education. Access to biologics decreased with age. Patients with PsA had odds ratios of access to biologics which were more than 50 percent higher, controlling for other variables. High disease severity, in terms of PASI ≥ 10 and DLQI ≥ 10, increased the odds ratios of access to biologics. Access to biologics was not influenced by BMI or by risk levels with respect to consumption of alcohol; however, smoking was negatively associated with access. With the exception of Southeast, all regions had lower odds ratios of access to biologics than Stockholm.

Results for the two alternative models were similar to the main results (See Supplementary Materials, Table S2). No statistically significant difference was detected with respect to educational levels, even when the closely related variable of income was excluded. When education was excluded, results for the income variables were similar to those in the main analyses.

In the subgroup analysis, including only patients without uncommon clinical types (*n* = 1811), the results were similar to those in the main analysis (See Supplementary Materials, Table S1). The results showed that higher incomes (fourth and fifth quintiles) were associated with higher odds ratios of receiving biologics (fourth quintile: OR = 1.46, 95% CI = 1.0–2.1; fifth quintile: OR = 1.96, 95% CI = 1.3–2.9) compared to the middle-income group.

## 4. Discussion

During the first decade of biologics for psoriasis, biosimilars, modern interleukin inhibitors and new small-molecule synthetics were not yet available. The landscape was more black-and-white, compared with today; there was a divide between treatment with biologics and nonbiological forms of treatment, mainly with methotrexate, which had been used since the 1960s. The difference in annual acquisition cost between biologics and methotrexate was close to one-hundred-fold.

This situation enabled a comprehensive analysis of the influence of socioeconomic factors on access to biologics, while controlling for disease severity, as well as clinical and demographic factors. The results showed that the higher-income group had better access to biologics than the middle-income group. The odds ratio of receiving biologics was almost twice as high, with an OR of 1.8 (CI 1.3–2.6), in the higher-income group, compared with the middle-income group. 

No previous published studies have investigated the influence of socioeconomic factors on the access to biologics in a Swedish setting, and we found only two other studies with a European setting, both from Italy [14,15].

This may be because such studies require data on both income and disease severity, and these may be difficult to acquire. As the level of severity varies greatly in psoriasis, merely administrative data are not sufficient. The present study was based on a unique dataset; it involved a relatively large sample of patients who were assessed in the course of everyday clinical practice, and included data on disease severity which was quantified by both the physician and the patient. In addition, the data were linked to a national administrative database of income and education, based on data from public authorities. 

The study reflects a period of nearly a decade, 2006–2014. In this time period access to biologics generally increased. We controlled for this effect by including years of observation. The earliest time period was associated with higher odds ratios of receiving biologics, compared with later time periods. This is most likely due to the fact that, in the early years of PsoReg registration, a higher proportion of patients on biologics were registered, compared with patients on nonbiologic treatments.

A limitation of this study is that the disease-severity measure PASI was developed for plaque psoriasis, and its use has been limited in less-frequent clinical types, such as forms of pustular psoriasis. However, in an alternative analysis, we excluded patients with uncommon clinical types, and the result, i.e., that patients in the fourth- and fifth-income quintiles had increased odds ratios of receiving biologics compared to the middle-income group, was robust. 

Another limitation is that there may have been selection- and omitted-variable bias. The selection towards patients on biologics, rather than those on systemic nonbiologic treatments, during the early years of patient inclusion in PsoReg may have contributed to over- or underestimation of the socioeconomic factors on the access to biologics. Furthermore, we did not have information about the patients’ residential municipalities. The finding of a significant result for income but not for education may have been influenced by differences in income and education between patients in rural and urban municipalities. 

The demographic pattern in this study was similar to that of previous studies based on earlier extractions of PsoReg data. Access to biologics increased with age [6], whereas no difference was detected with respect to differences in sex when controlling for disease severity [7]. Also, access to biologics was more strongly associated with high PASI values than DLQI values [18]. In an earlier study on regional differences [9], the odds ratios of receiving biologics during the entire time period were lower for the North, West and South regions, compared with the reference group Stockholm. In contrast to our study, no difference was found between the reference group and Uppsala. These differences may have resulted from differences in inclusion criteria, methods, and included variables. 

Differences between northern and southern regions might be explained by different levels of sun exposure, as the immunosuppressive effects of light and vitamin D could reduce the need for biologics [19,20]. However, our results revealed no such effects, as we failed to find any significant differences between north and south.

Similarly to our study, Scala and colleagues found that higher income was positively associated, and age negatively associated, with access to biologics in a recent observational study which included 18 Italian hospital centers [15]. As in our study, they found regional differences. However, and in contrast to our results, they also found that women were less likely to receive biologics than men, and that patients with a BMI ≥ 30 had lower odds of receiving biologics, compared with patients with BMI ≥ 25–30. The authors found that patients with PASI and DLQI ≥ 10 had lower odds of receiving biologics, compared to those with PASI and a DLQI < 10. This was due to the cross-sectional study design; in contrast to the patients in our study, the patients studied by Scala and colleagues were already using biologics, whereas our study controlled for pretreatment disease severity. While we found no significant differences in education, the findings of Scala and colleagues were inconsistent; patients with lower educational attainment (≤ junior high school) or a higher educational level (university or postgraduate) had lower odds of receiving biologics, compared to patients with a high school diploma.

Naldi and colleagues used the Italian Psocare register to investigate inequalities in access to biological treatments for psoriasis [14]. They found that, while controlling for disease severity and lifestyle factors, the number of prescriptions of biological agents increased with higher education and employment status of the patients. In our study, we did not see a statistically significant association with education, and employment status was not included; however, we did see an increased association with access to biologics in the highest income group. Similarly to our study, Naldi and colleagues found that increasing age, lower disease severity and smoking were negatively associated with access to biologics, whereas they found no difference in sex [14]. They also found geographical differences. Contrary to our results, they found that overweight and obese patients had increased odds ratios of receiving biologics, and that regular alcohol consumption was associated with lower odds ratios of receiving biologics. The higher odds ratios of receiving biologics among obese patients could possibly be due to development of a recalcitrant psoriasis form, which increases the need for biologics. 

Sweden has universal healthcare coverage, with limited private insurance and co-payments. As a result, income per se should not have a large impact on access to biologics. However, and as demonstrated in one Canadian study [21], universal healthcare does not guarantee equality. It can be hypothesized that the negotiation skills of patients in the high-income group (from the patient’s perspective) and the socioeconomic similarity between prescriber and high-income patient (from the prescriber’s perspective) results in increased access to the latest medical technologies. A socioeconomic gradient in access to biologics is not only a problem in terms of equity. It is also likely that the effectiveness of the drugs, and the associated health gains, are lower if patients with the highest incomes, rather than those with the greatest needs, obtain access to new medical technologies.

In the past couple of years, following the observation period covered in this study, biosimilars for the TNF inhibitors etanercept and adalimumab have become available. The introduction of biosimilars has created price pressures by increasing competition, leading to considerably lower treatment costs for biologics, both for biosimilars and the original products. This is likely to result in increased access for patients overall. Also, interleukin inhibitors have been introduced [22], making complete or almost-complete skin clearance an achievable treatment goal. Moreover, new synthetic treatment options have also become available. Future research should investigate whether, and to what extent, access to biologics has increased, and whether patients with lower severity of disease now obtain access to biologics, in a time period with lower acquisition prices. The extent to which the above-described socioeconomic gradient remains after the introduction of biosimilars, and, the extent to which it influences the access to interleukin inhibitors, remains to be seen. 

## 5. Conclusions

This study found that patients in the higher-income groups had better access to biologics than those in middle- and lower-income groups, even when adjusting for disease severity and other patient characteristics such as smoking and BMI. This may not only be an equity problem; it is also a matter of achieving the best possible health outcomes with society’s limited resources, as a better allocation of the resources may have resulted in a higher overall effectiveness of biologics.

## Figures and Tables

**Table 1:** Patient characteristics.

	Non-Biologics	Biologics	*p*-Value
*N*	1851	665	
Men, *n* (%)	1028 (56)	425 (64)	<0.001
Age, mean (SD)	55.3 (15.6)	46.8 (14.0)	<0.001
**Clinical type of psoriasis, *n* (%)**			
Plaque psoriasis	1512 (82)	586 (88)	<0.001
PsA	431 (23)	210 (32)	<0.001
Nail psoriasis	388 (21)	166 (25)	0.033
General Pustular Psoriasis	10 (<1)	9 (1)	0.038
Palmoplantar pustular psoriasis	106 (6)	12 (2)	<0.001
Palmoplantar Non pustular psoriasis	150 (8)	17 (3)	<0.001
Guttate psoriasis	51 (3)	20 (3)	0.736
Erythroderma	16 (<1)	16 (2)	0.002
Acrodermatitis *	11 (<1)	4 (<1)	0.981
**Disease severity**			
PASI, median (IQR) **	3.3 (1.5–6.7)	10.8 (6.3–15.8)	<0.001
DLQI, median (IQR) **	4 (1–8)	9 (4–17)	<0.001
Eligibility criteria, *n* (%)			
PASI < 10 & DLQI < 10	1138 (71)	169 (28)	<0.001
PASI ≥ 10 & DLQI < 10	104 (7)	141 (23)	
PASI < 10 & DLQI ≥ 10	250 (16)	97 (16)	
PASI ≥ 10 & DLQI ≥ 10	103 (6)	201 (33)	<0.001
**Lifestyle factors**			
BMI, mean (SD) **	27.7 (7.2)	28.3 (5.6)	0.055
Underweight	19 (1)	8 (1)	0.006
Normal weight	573(31)	180 (27)	
Overweight	714 (39)	233 (35)	
Obese	545 (29)	244 (37)	
Smoker, *n* (%) *	509 (28)	150 (23)	0.013
Risk alcohol, *n* (%) *	247 (13)	109 (16)	0.053
Regions **			
North	218 (12)	45 (7)	<0.001
Uppsala	195 (10)	80 (12)	
Stockholm	622 (34)	274 (42)	
Southeast	124 (7)	74 (11)	
South	327 (18)	86 (13)	
West	362 (19)	94 (15)	
**Year of inclusion, *n* (%)**			
2006–2008	138 (8)	90 (13)	<0.001
2009–2011	744 (40)	263 (40)	
2012–2014	969 (52)	312 (47)	
Education, *n* (%) **			
Low (≤9 years)	440 (24)	134 (20)	0.149
Medium (10–12 years)	927 (50)	348 (53)	
High (≥12 years)	473 (26)	181 (27)	
**Income**			
Disposable income, median (IQR) (in SEK1000)	2051 (1436; 2051)	2303 (1466; 2303)	0.031
Disposable income, *n* (%)			
1st Quintile	368 (20)	136 (20)	<0.001
2nd Quintile	401 (22)	103 (16)	
3rd Quintile	385 (21)	116 (17)	
4th Quintile	358 (19)	147 (22)	
5th Quintile	339 (18)	163 (25)	
Wage and business income, median (IQR) (in SEK1000)	1343 (0; 1343)	2468 (71; 2468)	<0.001
Wage and business income, *n* (%)			<0.001
1st Quintile	640 (34)	153 (23)	
2nd Quintile	161 (9)	53 (8)	
3rd Quintile	365 (20)	137 (21)	
4th Quintile	357 (19)	147 (22)	
5th Quintile	328 (18)	175 (26)	

BMI = Body Mass Index, DLQI = Dermatology Life Quality Index, IQR = Interquartile range, PASI = Psoriasis Area and Severity Index, PsA = Psoriatic Arthritis, Risk alcohol = High risk consumption of alcohol, SD = Standard Deviation. * Missing values which was assumed to be 0: Acrodermatitis = 1, Smoking = 16, Risk alcohol = 65, ** Missing values for which observation was excluded: PASI = 122, DLQI = 191, BMI = 77, Region = 15, Education = 13. T-tests for continuous variables, chi square test for categorical variables and Kruskal Wallis test for outcome measures of disease severity.

## Data Availability

The data that support the findings of this study were retrieved from PsoReg and Statistics Sweden. Under Swedish and European law, restrictions apply to the availability of these data, which were used under license for the current study, and so are not publicly available.

## Supplementary Materials

The following supporting information can be downloaded at: https://www.mdpi.com/article/10.3390/jcm12237234/s1, Table S1. Result of main regression model. Table S2: Result of alternative regression model specifications.

## Abbreviations

BMI	Body mass index
CI	Confidence interval
DLQI	Dermatology Life Quality Index
LISA	Swedish longitudinal database on health insurance and labour market studies
OR	Odds ratio
PASI	Psoriasis Area and Severity index
PsA	Psoriatic arthritis
PsoReg	Swedish Register for Systemic Treatment of Psoriasis
SD	Standard deviation
