# Supplementary material for: The Influence of Socioeconomic Factors on Access to Biologics in Psoriasis
Source: J Clin Med. 2023 Nov 22;12(23):7234. doi: 10.3390/jcm12237234 (PMC10707342; doi:10.3390/jcm12237234)
Supplement: Supplementary file 1 [file jcm-12-07234-s001.zip › jcm-2632999-supplementary.pdf]

Supplementary Materials

Table S1. Result of main regression model

**Access to Biologics**	**Odds Ratio**	**Std. Err.**	**z**	**P-value**	**[95%**	**CI]**
Age	0.97	0.00	-6.81	0.00	0.96	0.98
Female	0.90	0.12	-0.85	0.40	0.70	1.15
**Clinical Type of psoriasis**						
Psoriasis Arthritis	1.56	0.20	3.39	0.00	1.21	2.01
Nail psoriasis	1.16	0.16	1.07	0.29	0.88	1.53
**Disease Severity**						
PASI<10 & DLQI<10 (ref.)						
PASI ≥10 & DLQI <10	8.99	1.54	12.83	0.00	6.43	12.57
PASI <10 & DLQI ≥10	2.64	0.42	6.05	0.00	1.93	3.62
PASI ≥10 & DLQI ≥10	13.33	2.17	15.91	0.00	9.69	18.35
**BMI**						
Underweight	1.02	0.55	0.04	0.97	0.35	2.96
Normal weight (ref.)						
Overweight	0.97	0.14	-0.24	0.81	0.73	1.29
Obese	1.12	0.17	0.76	0.45	0.83	1.52
**Lifestyle factors**						
Smoker	0.66	0.09	-3.02	0.00	0.50	0.86
Risk alcohol	0.81	0.13	-1.27	0.21	0.59	1.12
**Year**						
2006-2008	1.76	0.34	2.91	0.00	1.20	2.57
2009-2011 (ref.)						
2012-2014	1.03	0.13	0.20	0.84	0.80	1.31
**Region**						
North	0.60	0.14	-2.23	0.03	0.39	0.94
Stockholm (ref.)						
Southeast	1.30	0.27	1.27	0.20	0.87	1.96
South	0.53	0.09	-3.56	0.00	0.38	0.75
Uppsala	0.62	0.12	-2.39	0.02	0.42	0.92
West	0.49	0.09	-4.04	0.00	0.35	0.69
**Income**						
1st Quintile	0.95	0.18	-0.28	0.78	0.65	1.37
2nd Quintile	0.94	0.19	-0.29	0.77	0.64	1.39
3rd Quintile (ref.)						
4th Quintile	1.42	0.26	1.93	0.05	0.99	2.04
5th Quintile	1.81	0.35	3.14	0.00	1.25	2.64
**Education**						
Low (≤9 years)	0.94	0.14	-0.38	0.71	0.70	1.27
Medium (10-12 years) (ref.)						
High (≥12 years)	0.92	0.13	-0.59	0.55	0.69	1.22
Constant	0.64	0.19	-1.46	0.15	0.36	1.16
Observations	2,124					

BMI= Body Mass Index, CI= Confidence Interval, DLQI= Dermatology Life Quality Index, PASI= Psoriasis Area and Severity Index, PsA=Psoriatic Arthritis, Risk alcohol= High risk consumption of alcohol

Table S2: Result of alternative regression model specifications

Access to Biologics	Main model	Alternative model 1 (only education)	Alternative model 2 (only income)	Subgroup analyses (excluding uncommon clinical types)
Age	0.97***	0.97***	0.97***	0.97***
Female	0.90	0.80*	0.91	0.93
**Clinical Type of psoriasis**				
Psoriasis Arthritis	1.56***	1.55***	1.57***	1.53***
Nail psoriasis	1.16	1.15	1.15	1.17
**Disease Severity**				
PASI <10 & DLQI<10 (ref.)				
PASI ≥10 & DLQI<10	8.99***	8.81***	8.90***	9.20***
PASI <10 & DLQI≥10	2.64***	2.52***	2.57***	2.63***
PASI ≥10 & DLQI≥10	13.33***	12.36***	13.31***	13.00***
**BMI**				
Underweight	1.02	0.98	1.05	1.03
Normal weight (ref.)				
Overweight	0.97	1.01	1.01	0.97
Obese	1.12	1.09	1.16	1.16
**Lifestyle factors**				
Smoker	0.66***	0.63***	0.66***	0.70**
Risk alcohol	0.81	0.82	0.83	0.78
**Year**				
2006-2008	1.76***	1.70***	1.79***	1.65**
2009-2011 (ref.)				
2012-2014	1.03	1.05	1.04	0.94
**Region**				
North	0.60**	0.58**	0.62**	0.61**
Stockholm (ref.)				
Southeast	1.30	1.27	1.33	1.24
South	0.53***	0.52***	0.54***	0.54***
Uppsala	0.62**	0.60**	0.63**	0.59**
West	0.49***	0.47***	0.50***	0.44***
**Income**				
1st Quintile	0.95		0.93	0.91
2nd Quintile	0.94		0.94	0.88
3rd Quintile (ref.)				
4th Quintile	1.42**		1.40*	1.46*
5th Quintile	1.81***		1.79***	1.96***
**Education**				
Low (≤9 years)	0.94	0.89		0.90
Medium (10-12 years) (ref.)				
High (≥12 years)	0.92	1.02		0.89
Constant	0.64	0.84	0.59*	0.65
Observations	2,124	2,124	2,136	1,811

*** p<0.01, ** p<0.05, * p<0.1

BMI= Body Mass Index, CI= Confidence Interval, DLQI= Dermatology Life Quality Index, PASI= Psoriasis Area and Severity Index, PsA=Psoriatic Arthritis, Risk alcohol= High risk consumption of alcohol
